# Characterization of genome-wide TFCP2 targets in hepatocellular carcinoma: implication of targets FN1 and TJP1 in metastasis

**DOI:** 10.1186/s13046-015-0121-1

**Published:** 2015-01-22

**Authors:** Xiao Xu, Zhikun Liu, Lin Zhou, Haiyang Xie, Jun Cheng, Qi Ling, Jianguo Wang, Haijun Guo, Xuyong Wei, Shusen Zheng

**Affiliations:** Division of Hepatobiliary and Pancreatic Surgery, Department of Surgery, First Affiliated Hospital, Zhejiang University School of Medicine, 79 QingChun Road, HangZhou, China; Key Lab of Combined Multi-Organ Transplantation, Ministry of Public Health, First Affiliated Hospital, Zhejiang University School of Medicine, HangZhou, China

**Keywords:** Hepatocellular carcinoma, Metastasis, Fibronectin 1, Tight junction protein 1, Transcription factor CP2

## Abstract

**Background:**

Transcription factor CP2 (TFCP2) is overexpressed in hepatocellular carcinoma(HCC) and correlated with the progression of the disease. Here we report the use of an integrated systems biology approach to identify genome-wide scale map of TFCP2 targets as well as the molecular function and pathways regulated by TFCP2 in HCC.

**Methods:**

We combined Chromatin immunoprecipitation (ChIP) on chip along with gene expression microarrays to study global transcriptional regulation of TFCP2 in HCC. The biological functions, molecular pathways, and networks associated with TFCP2 were identified using computational approaches. Validation of selected target gene expression and direct binding of TFCP2 to promoters were performed by ChIP -PCR and promoter reporter.

**Results:**

TFCP2 fostered a highly aggressive and metastatic phenotype in different HCC cells. Transcriptome analysis showed that alteration of TFCP2 in HCC cells led to change of genes in biological functions involved in cancer, cellular growth and proliferation, angiogenesis, cell movement and attachment. Pathways related to cell movement and cancer progression were also enriched. A quest for TFCP2-regulated factors contributing to metastasis, by integration of transcriptome and ChIP on chip assay, identified fibronectin 1 (FN1) and tight junction protein 1 (TJP1) as targets of TFCP2, and as key mediators of HCC metastasis. Promoter reporter identified the TFCP2-responsive region, and located the motifs of TFCP2-binding sites in the FN1 promoter, which then was confirmed by ChIP-PCR. We further showed that FN1 inhibition blocks the TFCP2-induced increase in HCC cell aggression, and that overexpression of TFCP2 can rescue the effects of FN1 inhibition. Knock down of TJP1 could also rescue, at least in part, the aggressive effect of TFCP2 knockdown in HCC cells.

**Conclusions:**

The identification of global targets, molecular pathways and networks associated with TFCP2, together with the discovery of the effect of TFCP2 on FN1 and TJP1 that are involved in metastasis, adds to our understanding of the mechanisms that determine a highly aggressive and metastatic phenotype in hepatocarcinogenesis.

**Electronic supplementary material:**

The online version of this article (doi:10.1186/s13046-015-0121-1) contains supplementary material, which is available to authorized users.

## Introduction

In the past decade hepatocellular carcinoma (HCC) has gone from being almost universally fatal, to a cancer that could be prevented, detected at an early stage and effectively treated [1,2]. However, due to the high incidence and inadequate surveillance, HCC remains the third most common cause of cancer-related mortality with about 600,000 patients dying annually from this aggressive disease around the world [3,4]. In China with the high prevalence of hepatitis B virus (HBV) infection, HBV-related HCC has become one of the main disease burdens. Intrahepatic or distant metastases at the time of diagnosis preclude many patients of the chance of surgery. Various factors such as microRNAs and transcription factors, contribute to metastasis by inducing HCC cell epithelial-mesenchymal transition (EMT), which is characteristic for the most aggressive metastatic cancer cells [5–8].

The transcription factor CP2 (TFCP2) has been shown to regulate diverse cellular and viral promoters, and plays roles in cell cycle progression and cell survival, as well as in cell lineage-specific functions, shown most strikingly to date in hematopoietic lineages [9–12]. TFCP2 is required for Snail1-induced Fibronectin (FN1) expression during EMT [13]. Yoo et al. [14] documented that TFCP2 overexpression is detected in more than 90% cases of human HCC patients compared to normal liver, and associated with the stage and grade of the disease. To better understand the molecular mechanisms by which TFCP2 contributes to cancer progression, researchers have identified and characterized several downstream target genes that are directly regulated by the TFCP2. These include osteopontin (OPN) and matrix metalloproteinase-9 (MMP-9) [14,15]. In addition, TFCP2 contributes to cell survival and cell cycle regulation by regulating the expression of thymidylate synthase (TS) [16]. Since TS is the target of 5-fluorouracil, TS up-regulation as a consequence of TFCP2 overexpression leads to 5-fluorouracil resistance [17]. The pivotal role of TFCP2 in hepatocarcinogenesis and chemoresistance indicates that TFCP2 inhibition might be an effective therapeutic approach for treating HCC. Therefore, the successful development of TFCP2 inhibitors will be potential for HCC chemotherapy but also rely on the clear mechanisms of TFCP2 signaling in HCC.

In this study, we identified TFCP2-related biological functions and pathways in HCC in different cellular contexts using Ingenuity Pathway Analysis (IPA). An integrated analysis of transcriptome and Chromatin immunoprecipitation (ChIP) on chip in HCC cells was used to screen for genes whose mRNA levels were regulated by TFCP2, and whose promoters were bound by TFCP2. We identified FN1 as a direct TFCP2 target, tight junction protein 1 (TJP1) as an indirect target, both as markers of EMT and key mediators of TFCP2 in HCC metastasis. This together with elucidation of direct targets of TFCP2 and genome-wide identification of targets and molecular pathways associated with TFCP2, will provide insights into its function and lead to better understanding of its role in hepatocarcinogenesis.

## Materials and methods

### Cell culture

Human HCC cell lines SMMC-7721, HepG2, Hep3B, SK-HEP-1, BEL-7402, Huh7, and MHCC-LM3 were purchased from Institute of Biochemistry and Cell Biology, Chinese Academy of Sciences (Shanghai, China) and maintained in Dulbecco’s modified Eagle’s medium (DMEM) or RPMI 1640 medium (Gibco, CA, USA) supplemented with 10% fetal bovine serum (Gibco, CA, USA) in a 37°C incubator with 5% CO_2_.

### Transfection with small interfering RNA and expression vectors

Negative control small interfering RNA (siRNA) and specific siRNA (siTFCP2: 5′-GCCAUUCCGAGUACAAAUATT-3′, siFN1: 5′-CUCUUGUGGCCACUUCUG ATT-3′) and siTJP1: 5′- GGGCUCUUGGCUUGCUAUUCGAATT-3′ were synthesized by Genepharma Corp (Shanghai, China). Cells were transfected with siRNA by Lipofectamine 2000 (Invitrogen, CA, USA). The HCC cells were used for real-time PCR, western blot, and gene chip after treating with siRNA for 48 h, used for migration and invasion after treating with siRNA for 24 h.

A hemagglutinin (HA) tag was inserted by restriction enzyme digestion onto the 3′ end of a TFCP2 cDNA clone. The resulting TFCP2-HA open reading frame was subcloned into lentivirus vector. The virus was produced in HEK293T transfected with either empty vector or the TFCP2-HA-containing construct along with the packaging helper plasmids, pRsv-Rev, MDLg/pRRE, pMD2.G. Virus was used to infect cells with 5 μg/mL polybrene. Stable cells were selected with 1 μg/mL puromycin in DMEM medium supplemented with 10% FBS.

### Cell proliferation assays, colony formation assays, and migration and invasion assays

Cell proliferation assays, colony formation, and migration and invasion assays were performed as described previously [18]. The assays were performed in triplicate after knockdown or overexpression of TFCP2 in the cells, respectively.

### Real-time PCR, western blotting

Real-time PCR was carried out using a ABI 7500 fast real-time PCR system as described previously [19]. Western blotting was performed and analyzed as described previously [18]. The primers for real-time PCR and the antibodies used for western blotting are listed in Additional file 1: Table S1 and Table S2, respectively.

### Gene chip analysis

Total RNA from HepG2 cells after knockdown of TFCP2 was isolated using Trizol (Invitrogen, CA, USA) according to a standard protocol, and was further purified with RNeasy Mini Kit (Qiagen, Valencia, CA, USA). A Roche NimbleGen Microarray (Human Expr 12x135K Arr Del) was used to analyze the transcriptome after the alteration of TFCP2. The siRNA experiment arrays were run in triplicate. Significantly up-regulated and down-regulated genes were defined by a 2-fold change from controls (*P* < 0.05 and FDR < 0.1).

### Chromatin immunoprecipitation (ChIP) on chip analysis

Antibodies that specifically immunoprecipitate TFCP2 are not currently available. We, therefore, established SK-HEP-1 cells that stably expressed TFCP2-HA for use in ChIP experiments. ChIP were performed using Chromatin Immunoprecipitation Kit (Merck Millipore) according to the instructions. The immunoaffinity-enriched DNA was proceeded for Nimblegen human promoter array assays or ChIP-PCR. The DNA isolated from ChIP with HA antibody from the TFCP2-HA-SK-HEP-1 cells, along with an input control, was amplified using linker-mediated PCR. Amplified DNA was labeled and hybridized to the Nimblegen human 3x720K RefSeq promoter array. The data from each hybridization was Z-score normalized, and the ratios of immunoprecipitation to input signal were determined. ChIPOTle analysis was performed for each array to identify peaks, using a sliding window approach.

### Construction of luciferase reporter plasmids

The human FN1 promoter region (−2000/+200) was obtained by PCR, using genomic DNA from BEL-7402 cells as a template. A series of 5′-deletion fragments of the FN1 promoter were generated by PCR using the primers shown in Additional file 1: Table S1. This was subcloned into KpnI (MluI)/XhoI sites of the pGL3-basic vector (Promega, Wisconsin, USA). All constructs were verified by sequencing.

### Promoter reporter assays

Cells were seeded in 24-well plates, grown to 60% confluence, and transfected using Lipofectamine 2000 according to manufacturer’s manual. In brief, each well was transfected with 400 ng of reporter constructs and 20 ng of pRL-TK plasmid (Promega, Wisconsin, USA) which was used to monitor transfection efficiency. At 48 h post-transfection, firefly luciferase activity was measured using a dual-luciferase reporter assay system (Promega, Wisconsin, USA). For siRNA transfection, cells were transfected with siRNA for 24 h and further transfected with plasmids.

## Results

### TFCP2 enhances HCC cell growth

**Figure 1:**
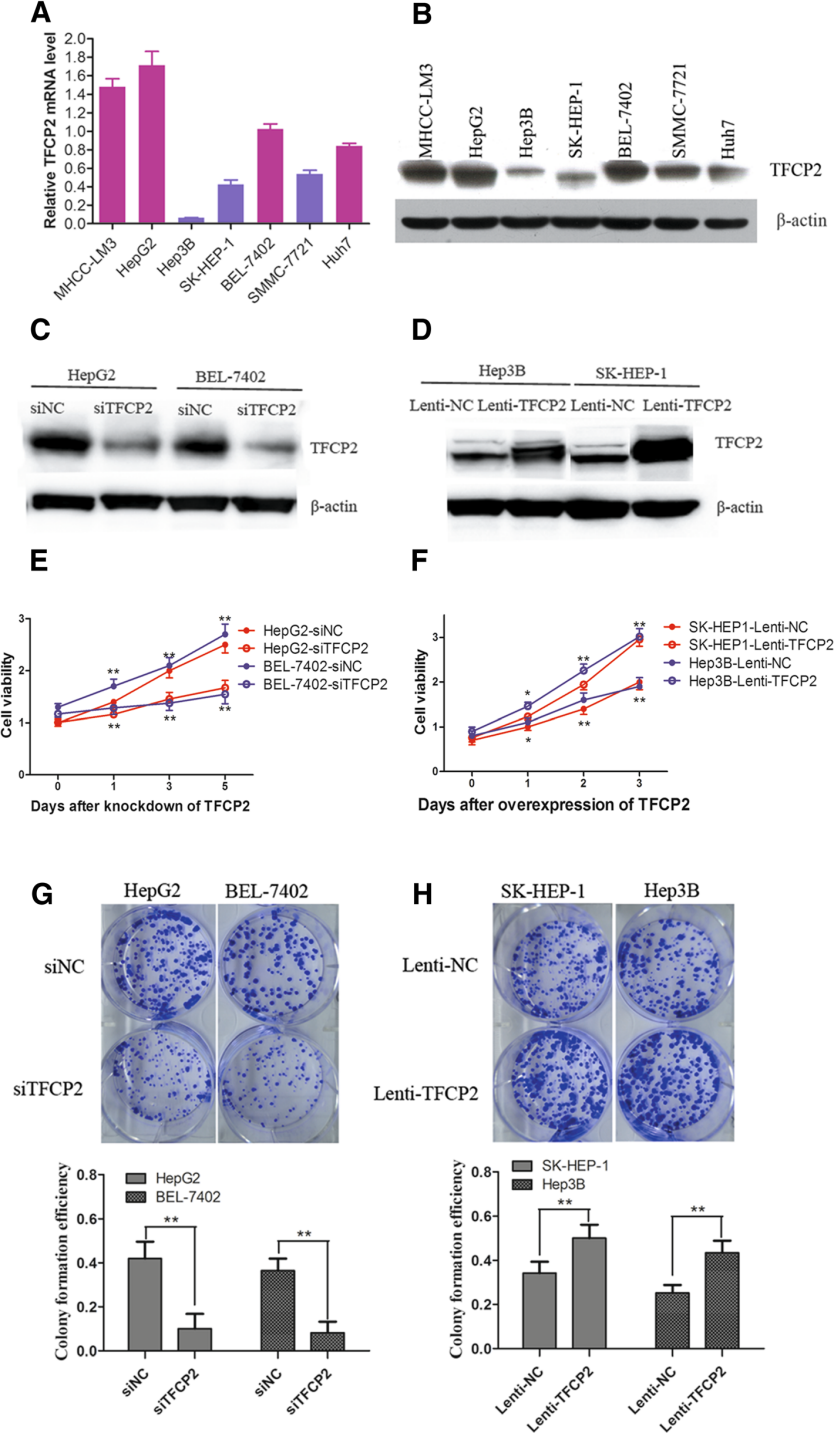
**TFCP2 promotes human HCC cell growth,** ***in vitro*** **. (A)** Real-time PCR and **(B)** western blotting for TFCP2 expression in human HCC cell lines, Red and blue bars represent high and low TFCP2 expression in HCC cells, respectively in **A.** β-actin as a loading control. **(C, D)** Western blotting of TFCP2 changes in HCC cells after siRNA transfection for 48 h or lentivirus infection. **(C)** Decreased TFCP2 protein in HepG2 and BEL-7402 cells transfected with siTFCP2 compared with negative control (siNC), **(D)** Increased TFCP2-HA fusion protein in Hep3B and SK-HEP-1 cells with lentivirus infection compared with negative control (Lenti-NC). **(E, F)** Cell proliferation assays in HCC cells with altered TFCP2 expression. The cell viability was determined by the CCK-8 assay in 96-well plates. All determinations were performed at least in seven replicates in three independent experiments. Values represent O.D. values mean ± SD (*P < 0.05, **P < 0.01; upper, HepG2 or SK-HEP1, lower, BEL-7402 or Hep3B). **(E)** HepG2 and BEL-7402 cells transfected with siRNA (F) Hep3B and SK-HEP-1 cells with lentivirus infection. **(G, H)** Colony formation assays in HCC cells with altered TFCP2 expression in cell culture plates. Representative photographs of colony formation from different cell lines are shown in the upper panels. The colony formation rate is shown in the lower panels (calculated by dividing the colony numbers by the plated cells). Data were obtained from three independent experiments. **(G)** HepG2 and BEL-7402 cells transfected with siRNA for 24 h and then used for colony assays, **(H)** Hep3B and SK-HEP-1 cells with lentivirus infection (**P < 0.01).

TFCP2 protein expression was detected in human HCC cell lines by real-time PCR and western blotting (Figures 1A and B). The results showed that HepG2, BEL-7402, and MHCC-LM3 were associated with high TFCP2 expression, and Hep3B and SK-HEP-1 cell lines were characterized by low TFCP2 expression. To determine the effect of TFCP2 on HCC growth and aggressiveness, we constructed human HCC cell lines (HepG2 and BEL-7402) with depleted TFCP2 using siRNA. We also constructed cell lines (Hep3B and SK-HEP-1) with stable TFCP2-HA overexpression using lentivirus. Successful alterations of TFCP2 expression in these cells were confirmed by western blotting (Figures 1C and D).

Knockdown of TFCP2 significantly inhibited tumor cell growth in HepG2 and BEL-7402 cells compared with the control cells. By contrast, overexpression of TFCP2 significantly increased the growth of Hep3B and SK-HEP-1 cells (Figures 1E and F). Colony-formation assays were performed in cells with altered TFCP2 expression. As shown in Figures 1G and H, knockdown of TFCP2 resulted in significantly lower colony formation efficiency in HepG2 (10.12 ± 6.6% *vs* 42.25 ± 7.8%) and BEL-7402 cells (8.20 ± 5.0% *vs* 36.50 ± 5.4%) relative to control cells (*P* < 0.01). By contrast, TFCP2 overexpression resulted in significantly higher colony formation efficiency in SK-HEP-1 and Hep3B cells than in control cells (*P* < 0.01). These findings suggest that TFCP2 signaling enhances the colony formation capacity of HCC cells.

### TFCP2 enhances HCC cell aggressiveness

**Figure 2:**
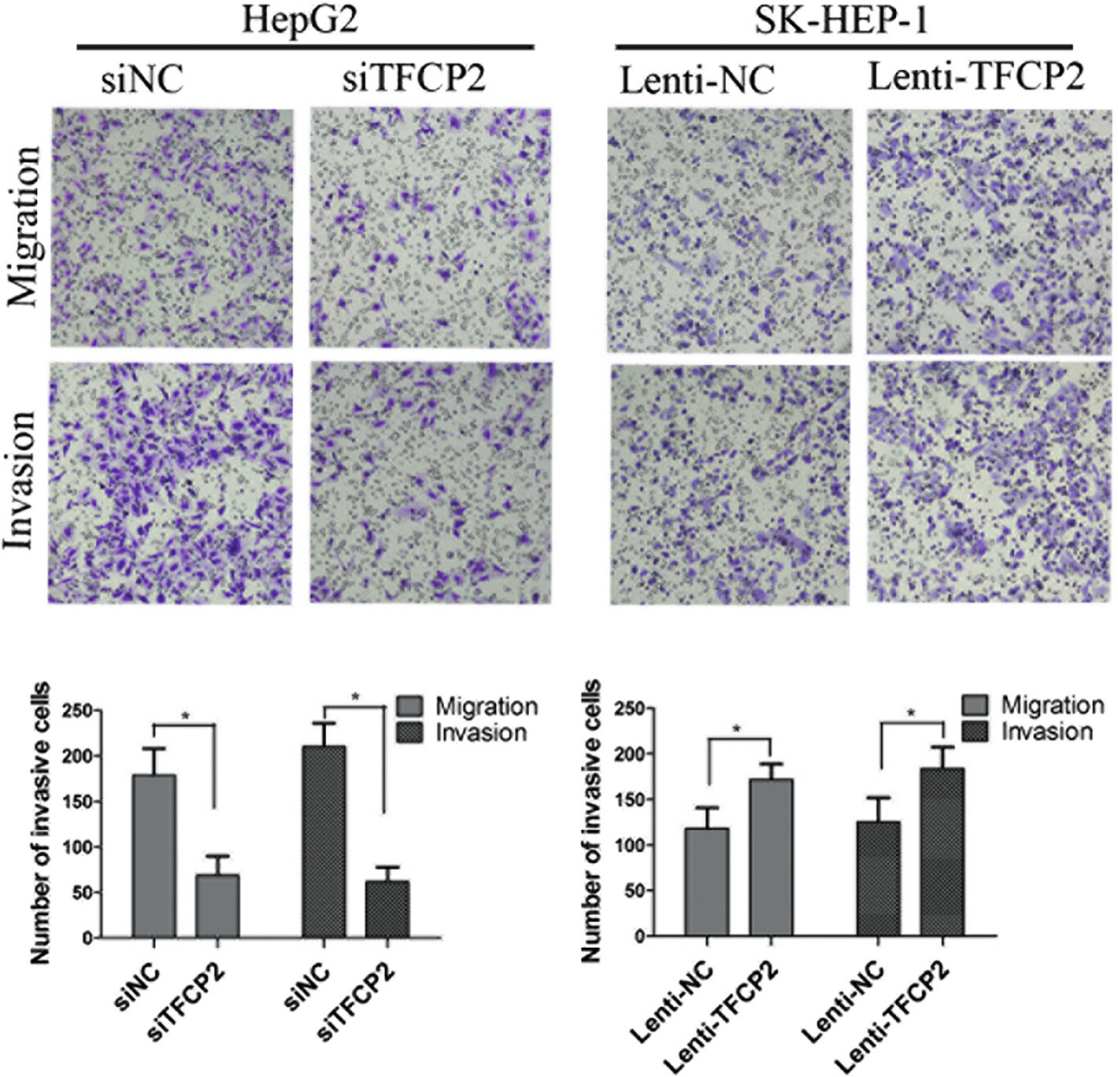
**The effect of TFCP2 on cell migration and invasion.** The TFCP2 knockdown in HepG2 significantly inhibited the migration and invasion of HCC cells compared with controls, HepG2 transfected with siRNA for 24 h and then used for miagration and invasion. Whereas TFCP2 overexpression in SK-HEP-1 promoted migration and invasion. The migration and invasion assays were assessed at 24 and 48 h, respectively. Representative fields of invaded cells from independent groups are shown in the upper panels. Quantitative analysis of invaded cells is shown in the lower panels. The data are presented as mean ± SD of three different experiments (*P < 0.01).

Since migratory and invasive behaviors are indicators of the metastatic potential, we performed migration and invasion assays to examine the impact of TFCP2 on HCC cell aggressiveness. The TFCP2-knockdown HepG2 cells migrating toward the conditioned medium or invading through the matrigel were significantly lower than controls in the migration (69.1 ± 21.2 *vs* 179.4 ± 28.7) and invasion assay (62.4 ± 16.1 *vs* 210.3 ± 26.5). They were higher than controls in TFCP2-overexpressed SK-HEP-1 cells. The results are shown in Figure 2. Similar changes in cell aggressiveness pattern were observed in BEL-7402 or Hep3B cells after corresponding alterations of TFCP2 (Additional file 2: Figure S1). These findings suggest that TFCP2 may be an important contributor to the migration and invasion of HCC cells.

### Genes and biological functions that respond to TFCP2 alteration in HCC cells

Gene expression array analysis was performed in HepG2 cells after knockdown of TFCP2. Statistical analysis performed in the transcriptome dataset showed that in HepG2, 454 genes were significantly down-regulated and 357 genes up-regulated after knockdown of TFCP2 (>2.0-fold change). We validated these data using real-time PCR in nine genes randomly obtained after alteration of TFCP2 in HepG2, representing all three expression patterns (up-regulated, unchanged, and down-regulated). Good concordance was observed between the results from real-time PCR and those from the cDNA array (Additional file 2: Figure S2). The complete data from the transcriptome analysis is shown in Additional file 3: Table S3. IPA was undertaken to identify TFCP2-relevant biological functions from differentially expressed genes in each cell line. The top 7 significant functional classification of TFCP2-regulated genes, ranked by *P*-value are shown in Table 1. Notably, among these significant functions, growth, adhesion, binding, and angiogenesis were observed in HepG2 after TFCP2 knockdown.

**Table 1:** **Bio-function analysis of the TFCP2-regulated genes in HepG2 knock down of TFCP2**

**Category**	**Functions**	***P*** **-Value**	**Activation State** ^**†**^	**z-score** ^**†**^	**#Molecules** ^**§**^
Cancer	Growth	2.81E-07		−0.508	28
Cellular assembly and organization	Adhesion	5.47E-07			11
Cell-To-Cell Signaling and Interaction	Binding	1.15E-06	Decreased	−2.412	6
Cardiovascular System Development and Function	Angiogenesis	1.22E-05	Decreased	−2.297	32
Cellular Movement	Cell movement of carcinoma cell lines	6.24E-05	Decreased	−2.252	11
Cell-To-Cell Signaling and Interaction	Attachment of cells	1.93E-04	Decreased	−2.412	7
Cellular Movement	Chemotaxis of tumor cell lines	1.17E-03	Decreased	−2.036	10

^**†**^Activation State & z-score, z-score was calculated by the IPA z-score algorithm, reflecting the predicted direction of change for the function, an absolute z-score of ≥ 2 is considered significant. Activation State is: Increased if the z-score is ≥ 2, Decreased if the z-score ≤ −2, Blank (empty) - IPA does not make a prediction for the process or disease. ^§^The number of molecules (genes) that are associated with each function.

### Identification of TFCP2 direct target genes by ChIP on chip

We used a ChIP on chip assay in TFCP2-HA-SK-HEP-1cells, using the 3x720K RefSeq promoter array (NimbleGen), to identify genes that were directly regulated by TFCP2. Our results showed that a total of 1095 promoters were enriched by ChIP on chip (FDR ≤0.01, peak-score >0.50) (Additional file 4: Table S4). Interestingly, the 1095 direct targets were most enriched from gene functions involved in cancer, gastrointestinal disease and respiratory disease. Other prominent functions were cell-to-cell signaling and interaction, drug metabolism, embryonic and organism development, and cell morphology (Additional file 2: Figure S3). By integrating TFCP2-ChIP targets with the TFCP2-regulated transcriptome, 25 overlapped genes are listed in Additional file 1: Table S5, which also contains the results of validation by real-time PCR with 4 cell lines.

**Figure 3:**
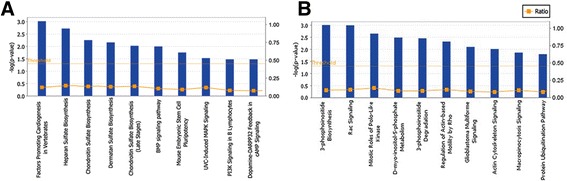
**Signal pathways of the TFCP2 target genes.** Microarray and ChIP on chip data grouped by signal pathways ranked in order of statistical significance. The ratio of genes (orange line) refers to number of genes involved in a pathway divided by total genes. Data and images were generated using Ingenuity Pathways Analysis. The most important signal pathways were based on ChIP on chip data in SK-HEP-1 cells **(A)**, and gene array data in HepG2 after knockdown of TFCP2 **(B)**.

IPA was used to identify the signal pathways associated with the TFCP2-ChIP targets in TFCP2-HA-SK-HEP-1and TFCP2-regulated genes from gene arrays in HepG2. Based on ChIP on chip in TFCP2-HA-SK-HEP-1cells, the most important signal pathways were factors promoting cardiogenesis, heparin sulfate biosynthesis, and chondroitin sulfate biosynthesis (Figure 3A). In HepG2 cells, 3-phosphoinositide biosynthesis, rac signaling, and mitotic roles of polo-like kinase were identified as important pathways after knockdown of TFCP2 (Figure 3B). Pathways 6, and 8 based on ChIP on chip in TFCP2-HA-SK-HEP-1 were associated with cancer, and pathways 2, 6, and 8 in HepG2 cells were associated with cell adhesion and motility related pathways. This finding supports a possible role of these newly identified targets of TFCP2 in HCC progression and other biological processes.

Networks were created using IPA to interpret differentially expressed gene interactions in HCC cells. The scores, derived from *P*-values, indicated the likelihood of focus genes belonging to a network. In HepG2 cells, the top three networks (scores = 34, 34, 30, respectively) were involved in protein synthesis/degradation, cellular assembly, cancer, and cell cycle (Additional file 2: Figure S4).

### TFCP2 signaling in migration/invasion of HCC cells

**Figure 4:**
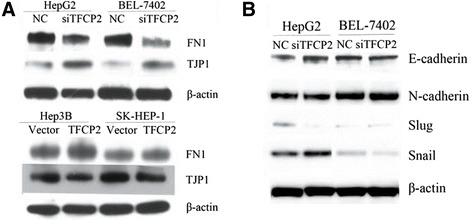
**TFCP2 positively regulates FN1 and negatively regulates TPJ1 in human HCC cells.** FN1 and TJP1 protein expression was detected in HepG2 and BEL-7402 cells after TFCP2-knockdown (treated with siTFCP2 for 48 h) and in Hep3B and SK-HEP-1 cells after TFCP2-overexpression **(A)** by Western blot analysis. Other markers of EMT (E-cadherin, N-cadherin, slug, and snail) were not obvious regulated by TFCP2 **(B)**.

To test which genes mediated TFCP2-promoting migration/invasion of HCC, we focused on the markers of EMT by integrating TFCP2-ChIP targets with the TFCP2-regulated transcriptome (Additional file 1: Table S5). Among the common genes identified by both TFCP2-ChIP targets and TFCP2-regulated transcriptome, FN1 and TJP1 were selected for further characterization on the basis of their relatively high binding intensity in the ChIP on chip and the fact that they are important makers of EMT that mediates the migration/invasion of different cancer cells [20,21]. In accordance with FN1 and TJP1 roles in aggressive metastatic cancer cells, the former promotes and the latter suppresses the cancer cell aggression, western blotting demonstrated that FN1 was positively regulated and TJP1 was negatively regulated by TFCP2 in human HCC cells (Figure 4A). Furthermore, we also validated whether other markers of EMT were indirectly regulated by TFCP2, as they were not enriched in ChIP on chip. As showed in Figure 4B, N-cadherin, E-cadherin, slug, and snail were not affected by TFCP2.

### TFCP2 activates the FN1 promoter

**Figure 5:**
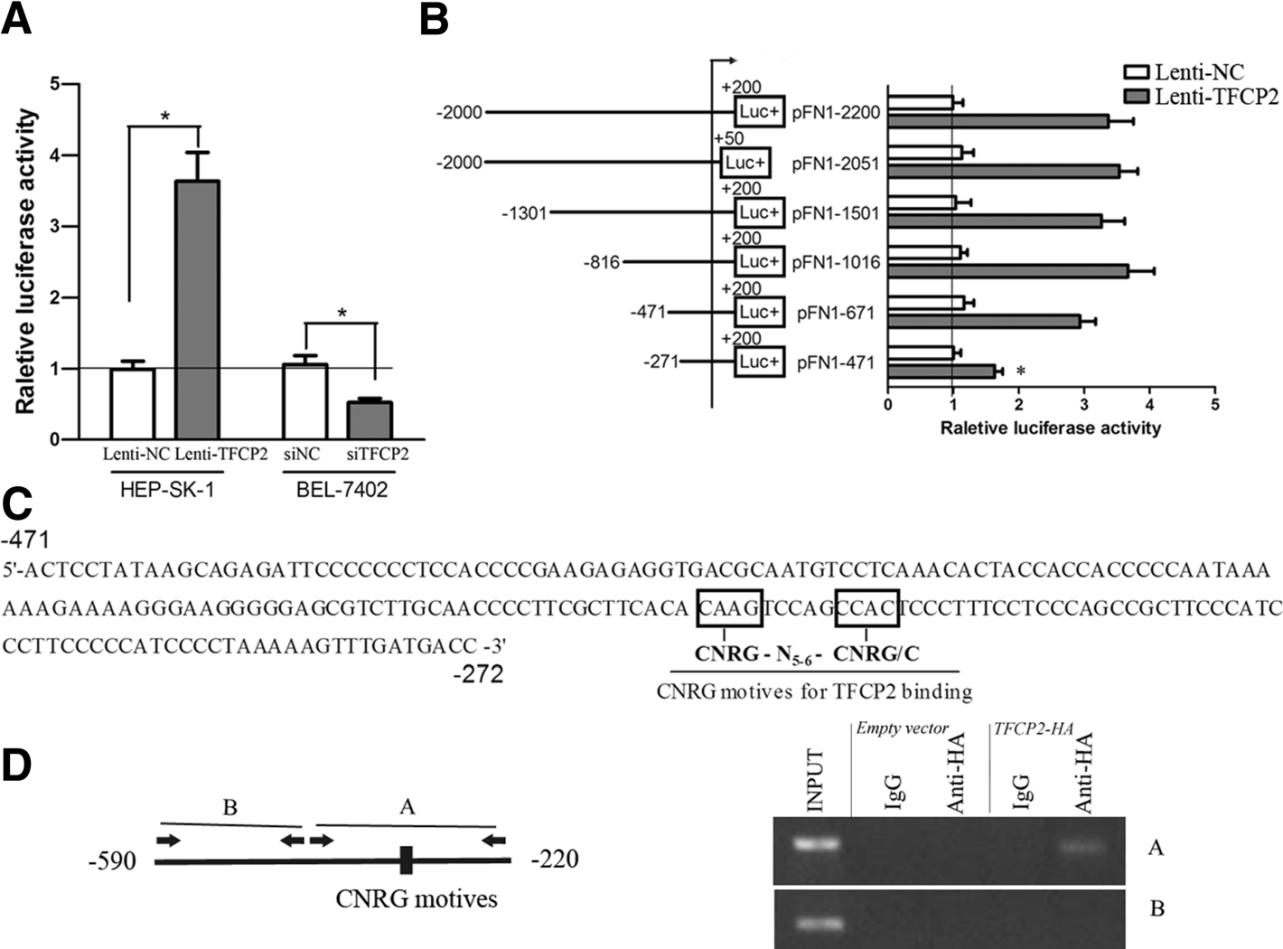
**Deletional analysis of the FN1 minimal promoter, and analysis of TFCP2-binding sites. (A)** FN1 promoter activity in SK-HEP-1 and BEL-7402 cells after the alteration of TFCP2. Firefly luciferase activity representing FN1 promoter activity was measured after transfection with pFN1-2200 and normalized to Renilla luciferase activity. **(B)** Schematic representation of the FN1 promoter (and its fragmental constructs) and transcription activation of FN1 promoter fragments by TFCP2. SK-HEP-1 control and overexpression cells were transfected with the deletion fragmental constructs of the FN1 promoter and renilla TK plasmid for normalization. All determinations were performed at least in triplicate in three independent experiments. Values represent mean ± SD. *P < 0.01. **(C)** The −471/-272 sequence of the FN1 promoter includes CNRG-N_5–6_-CNRG/C, as a putative site for TFCP2 binding. The positions of potential regulatory motifs are indicated by rectangles. Consensus sequence is annotated, where N = T, C, A or G; and R = A or G. **(D)** Schematic diagram of FN1 promoter showing the location of potential TFCP2-binding sites in the promoter and primers designed for ChIP assay. Fragment A and B represent fragments with potential TFCP2-binding consensus or without, respectively. ChIP using anti-IgG or anti-HA were performed with stable SK-HEP-1 cell lines expressing TFCP2-HA or not (empty vector) followed by PCR using primers to the candidate target promoter regions of FN1.

To explore molecular mechanisms by which TFCP2 induces FN1 expression, we cloned ~2 kb region of human FN1 gene starting from −2000 to +200, where the translation start site was regarded as +1. FN1-Prom-luc (−2000/+200) was transfected into cells together with a renilla TK plasmid. Firefly luciferase activity was normalized by renilla luciferase activity. TFCP2-overexpression significantly increased FN1 promoter activity in SK-HEP-1 cells, and TFCP2-knockdown significantly decreased FN1 promoter activity in BEL-7402 cells (Figure 5A). The same promoter assay was used to TJP1, however TFCP2 didn’t significantly change the TJP1 promoter activity (data not shown). TJP1 may be regulated indirectly by TFCP2. Next, we selected FN1 for further characterization.

To delineate the region(s) of the FN1 promoter that were induced by TFCP2, we attempted to delete the promoter region (Figure 5B). Successive 5′-deletions from positions −2000 to −471 resulted in, at most, moderate changes in FN1 promoter activity induced by TFCP2 in SK-HEP-1. However, elimination of the region from-471 to −272 resulted in a marked decrease in TFCP2-induced FN1 promoter activity, indicating that the four potential TFCP2 binding elements between −471 and −271 contribute to TFCP2 responsiveness. Analysis of this FN1 promoter region identified the potential TFCP2-binding consensus of four base motifs separated by a linker of five to six bases (CNRG-N_5–6_-CNRG/C) (Figure 5C). This is consistent with reports for other members of the CP2 family [22,23]. As such we designed two sets of PCR primers around the promoter between −471 and −271, the first pair flanking the TFCP2 potential binding sites (fragment A), while the other pair not (fragments B), to confirm direct binding of TFCP2 to FN1 promoter by ChIP. ChIP assay demonstrated TFCP2 binding only to the proximal part with TFCP2-binding sites and not to the distal part without (Figure 5D). These results confirm that TFCP2 transcriptionally up-regulates FN1.

### FN1 and TPJ1 mediate the TFCP2-induced increase in HCC cancer cell aggression

**Figure 6:**
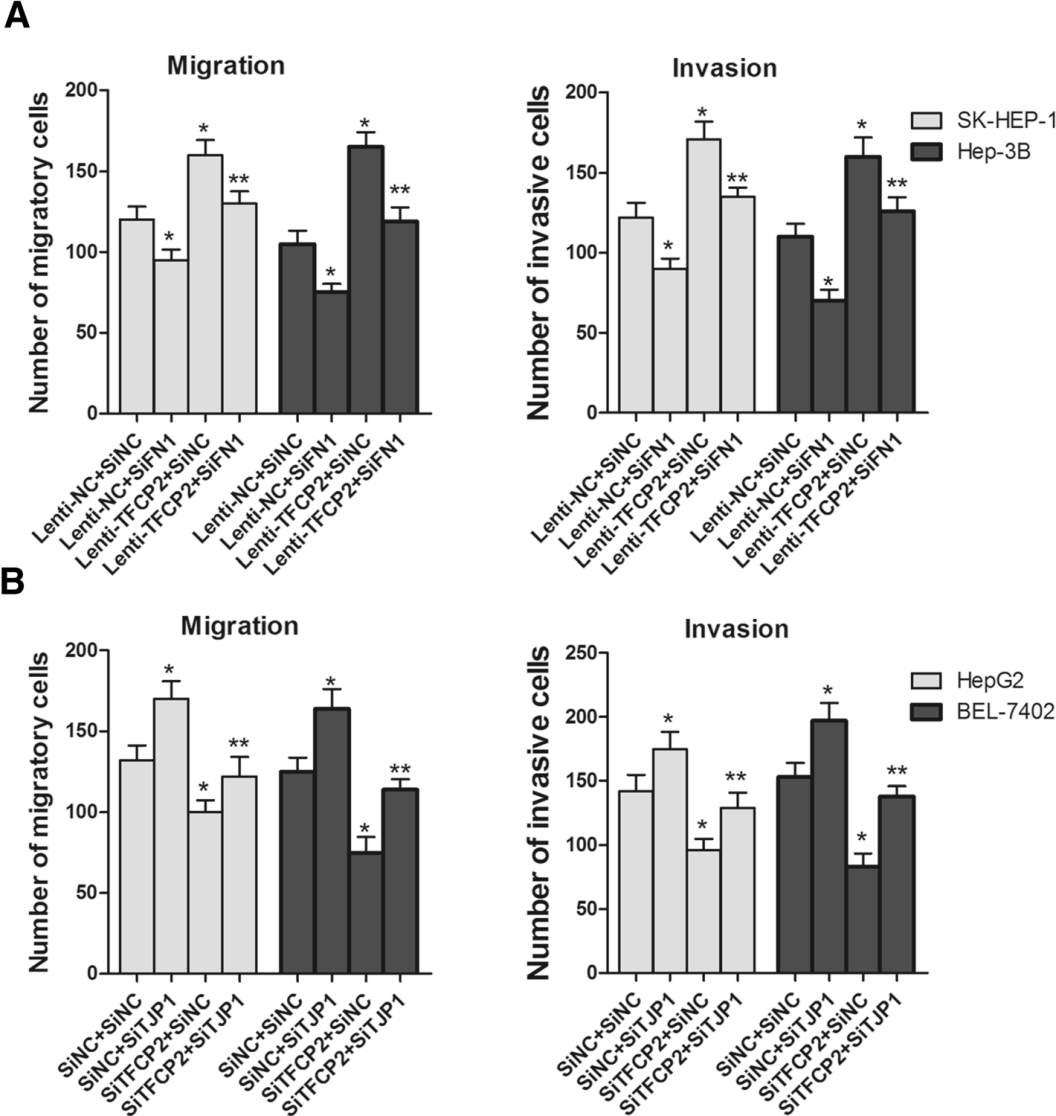
**TFCP2 promotes migration/invasion through up-regulating FN1 and down-regulating TJP1. (A)** FN1 inhibition partially reverses the aggressive effect by TFCP2 overexpression in SK-HEP-1 and Hep3B cells. Transient transfection with FN1 siRNA results in a significant reduction in migratory and invasive behaviors analyzed by migration and invasion assays (*, P < 0.05 compared with SiNC). Cells infected by TFCP2 vectors and transiently transfected with FN1 siRNA showed no increase in aggression by TFCP2 overexpression (**, P > 0.05 compared with cells with Lenti-NC + SiNC). **(B)** SiTJP1 could also rescue, at least in part, the aggressive effect of TFCP2 knockdown in HepG2 and BEL-7402 cells. Transient transfection with TJP1 siRNA results in a significant increase in migratory and invasive behaviors analyzed by migration and invasion assays (*, P < 0.05 compared with SiNC). Cells transfected by si-TFCP2 and TJP1 siRNA showed no reduction in aggression by si-TFCP2 (**, P > 0.05 compared with cells with SiNC + SiNC).

As FN1 is a downstream activation target of TFCP2, we predicted that FN1 inhibition could block the aggressive effect caused by overexpression of TFCP2. To test this prediction, we treated cells with FN1 siRNA with or without TFCP2 overexpression. Cells infected by TFCP2 vectors and transiently transfected with FN1 siRNA showed no increase in aggression by TFCP2 overexpression. As compared to transfection with the negative control, transfection with the FN1 siRNA resulted in significant protection against the aggressive effect of TFCP2 overexpression (Figure 6A). The same rescue experiments was performed for TJP1.Transient transfection with TJP1 siRNA results in a significant increase in migratory and invasive behaviors analyzed by migration and invasion assays. Cells transfected by SiTFCP2 and TJP1 siRNA showed no reduction in aggression by SiTFCP2 (Figure 6B). The above results suggest that FN1 and TJP1 mediate, at least in part, the aggressive effect of TFCP2 signal.

## Discussion

TFCP2 is a ubiquitously expressed transcription factor in mammalian cells [9]. Substantially enhanced expression of TFCP2 in hepatocellular carcinoma has been shown to promote oncogenesis [14,24]. In agreement with previous findings, we demonstrated that TFCP2 was critical in maintaining cellular matrigel invasion, proliferation and metastasis [14]. In order to identify TFCP2 binding targets on a genome-wide scale, we performed ChIP for TFCP2 in a human HCC cell line SK-HEP-1. Additionally, a global gene expression analyses was performed in HCC cells HepG2 after down-alteration of TFCP2. We investigated a comprehensive transcriptional network of genes under the control of the transcription factor TFCP2 in HCC cell lines, and identified the direct targets of TFCP2, as well as a number of indirect targets that are affected TFCP2. Furthermore, FN1 and TJP1 were identified as the TFCP2 target genes, mediating TFCP2-induced metastasis in HCC.

IPA analysis shows that biological functions involving growth, adhesion, binding, and angiogenesis and so on are significantly enriched in the TFCP2 transcriptome in HepG2 cells. As regard TFCP2-ChIP targets in SK-HEP-1, the functions associated with cancer, cell movement, cell cycle, cell-to-cell signaling and interaction, cellular growth and proliferation were also significantly enriched. These findings indicate that although cell lines from different lineages can share common TFCP2-regulated biological functions, the genes that are involved could be quite distinct.

TFCP2 augments HCC progression and metastasis by altering multiple physiologically important pathways. IPA analysis of the direct and indirect targets of TFCP2 identified the pathways affected by changes in the expression level of TFCP2 target genes. Interestingly, unlike the biological functions, there are few common pathways shared by the TFCP2-regulated genes in the two cell lines. Among the significant pathways identified by ChIP on chip or microarrays, most based on ChIP on chip in SK-HEP-1 cells were associated with growth and development, and the extracellular matrix. The pathways in HepG2 cells after TFCP2 knockdown were associated with cell adhesion and motility. These findings support a possible role for these newly identified targets of TFCP2 in HCC progression and other biological processes.

Target genes of transcription factors are known to be highly context- and cell type- specific. In this study, we used SK-HEP-1 cells as an *in-vitro* screening tool for identifying specific targets of TFCP2 binding in HCC. While we believe that the majority of identified TFCP2 target genes in SK-HEP-1 cells are likely to be present in other HCC cells. It is also plausible that binding targets that require TFCP2 and additional co-factors may not be fully represented in our system. Because unique co-factors present in distinct cell types are likely to play a significant role in dictating DNA binding specificity and/or affinity of the TFCP2 transcription complex.

Our experiments also established the presence of direct transcriptional regulation of FN1 by TFCP2. FN1 is a typical mesenchymal gene involved in EMT [25]. Recent studies have shown that FN1 can bind to collagen/gelatin, heparin, and cell surface receptors, and that it plays an important role in cell adhesion, migration, and differentiation [26,27]. It has also been shown that FN1 down-regulation suppresses the migration and invasion [28,21]. Therefore, the demonstration that FN1 is a direct downstream target of TFCP2 establishes a new molecular wiring which may, at least partially, explain how TFCP2 amplification contributes to HCC progression and metastasis. Additionally, TJP1, as the TFCP2 indirect target, mediates the TFCP2-promoting HCC progression. Our findings also showed that the core promoter was found from the human FN1 promoter region (−2000 to +200), and contained the putative regulatory motifs (CNRG-N_5–6_-CNRG/C). Inconsistent with the previous report [13], we found the TFCP2 regulation on FN1 through the same motifs. This further illustrates that TFCP2 regulates FN1 across different cell systems. Porta-de-la-Riva et al. [13] found that FN1 was up-regulated upon EMT induction by Snail1 in normal development, then TFCP2 was found to bind the FN1 proximal promoter, which responses to Snail1. In our paper, FN1 was selected from ChIP on chip and array results of TFCP2 in cancer (HCC), we used a series of 5′-deletion fragments of the FN1 promoter (~2 kb region) to locate the responsive minimal promoter to TFCP2 and found involvement of TFCP2 in migration, as mediated through its direct upregulation of FN1.

In conclusion, our data provide a comprehensive list of the direct targets of TFCP2, and identify a number of indirect targets that are affected by loss or gain of TFCP2. We also investigated the transcriptional and molecular networks and associated pathways in HCC cells as well as the effect of TFCP2 on FN1 and TJP1, major player of HCC metastasis as well as key regulator of mesenchymal cell phenotype. These findings provide a molecular foundation for future studies aimed at understanding the mechanisms of TFCP2 signaling in hepatocarcinogenesis. They may also facilitate the design of TFCP2-based cancer therapy. Indeed, the availability of small molecule inhibitors of TFCP2 may herald a novel generation of anti-HCC agents with significant therapeutic potential and encourage further research into the role of TFCP2.

## Additional files

Additional file 1: Table S1. The primer sequences. **Table S2.** List of antibodies. **Table S5.** Integrate TFCP2-ChIP targets with the TFCP2-regulated transcriptome. Additional file 2: Figure S1. The effect of TFCP2 on cell migration and invasion. The TFCP2 knockdown in BEL-7402 significantly inhibited the migration and invasion of HCC cells compared with controls, whereas TFCP2 overexpression in Hep3B promoted migration and invasion. The migration and invasion assays were assessed at 24 and 48 h, respectively. The data are presented as mean ± SD of three different experiments (**P* < 0.01). **Figure S2.** Confirmation of microarray results by qRT-PCR. Changes of selected mRNAs between the alteration of TFCP2 versus NC in HepG2 after siTFCP2. **Figure S3.** Biological function analysis of the TFCP2-ChIP targets in SK-HEP-1. **Figure S4.** Network analysis of molecule interactions from microarray data in HepG2 cells. Knowledge-based IPA network using genes in HepG2 siTFCP2-signature (genes are shown in Additional file 3: Table S3). Green/red: genes down/up-regulated in HepG2 after knockdown TFCP2. Solid lines: direct interactions, and dashed lines: indirect interactions. Additional file 3: Table S3. The transcriptome analysis in HepG2 after knockdown of TFCP2. Additional file 4: Table S4. ChIP on chip assay in SK-HEP-1 cells to identify genes that were directly regulated by TFCP2.

## Abbreviations

TFCP2: Transcription factor CP2
FN1: Fibronectin 1
TJP1: Tight junction protein 1
MMP-9: Matrix metalloproteinase-9
TS: Thymidylate synthase
HBV: Hepatitis B virus
HCC: Hepatocellular carcinoma
EMT: Epithelial-mesenchymal transition
IPA: Ingenuity Pathway Analysis
HA: Hemagglutinin
ChIP: Chromatin immunoprecipitation
