# Supplementary material for: Characterization of genome-wide TFCP2 targets in hepatocellular carcinoma: implication of targets FN1 and TJP1 in metastasis
Source: J Exp Clin Cancer Res. 2015 Jan 22;34(1):6. doi: 10.1186/s13046-015-0121-1 (PMC4311423; doi:10.1186/s13046-015-0121-1)
Supplement: Additional file 1: Table S1. — The primer sequences. Table S2. List of antibodies. Table S5. Integrate TFCP2-ChIP targets with the TFCP2-regulated transcriptome. [file 13046_2015_121_MOESM1_ESM.doc]

**Table S1.** The primer sequences

| Primer sequences for real-time PCR | |
| --- | --- |
| GAPDH forward | ATGGGGAAGGTGAAGGTGG |
| GAPDH reverse | GGGGTCATTGATGGCAACAATA |
| TFCP2 forward | CGAGTTTGCCTCCTGAT |
| TFCP2 reverse | GTCCTTGATTGAGATACGTTAG |
| FN1 forward | TGCTGGGACTTCCTATGTG |
| FN1 reverse | CCTCGATTATCCTTCTTGC |
| primer sequences for constructing luciferase reporter plasmids | |
| FN1-471-KpnIF | cggggtaccGCAAAGGAAACCGAAAAAAAG |
| FN1-471-XhoIR | ccgctcgagCAACCTGCGGGAAAAATCC |
| FN1-671-KpnIF | cggggtaccACTCCTATAAGCAGCAGAGATTCCCC |
| FN1-671-XhoIR | ccgctcgagCAACCTGCGGGAAAAATCC |
| FN1-1016-KpnIF | cggggtaccCTTGCAAAAGAAAACTTCATTC |
| FN1-1016-XhoIR | ccgctcgagCAACCTGCGGGAAAAATCC |
| FN1-1501-KpnIF | cggggtaccCAAAAAGGACTGATGTGATGTG |
| FN1-1501-XhoIR | ccgctcgagCAACCTGCGGGAAAAATCC |
| FN1-2051-MluIF | cgacgcgtATGGTTTTGGTTGACCAAATACTTTTC |
| FN1-2051-XhoIR | ccgctcgagCGCGCCTGGGGTTCCCTC |
| FN1-2200-MluIF | cgacgcgtATGGTTTTGGTTGACCAAATAC |
| FN1-2200-XhoIR | ccgctcgagCAACCTGCGGGAAAAATCC |
| primer sequences for ChIP PCR | |
| FN1 A forward | ACTCCTATAAGCAGAGATTC |
| FN1 A reverse | CCTTTGCGGTCATCAAACT |
| FN1 B forward | GTACCCTTTAGTCCAAAGA |
| FN1 B reverse | GTGAGGAAGAAAACCCATA |

**Table S2.** List of antibodies

| **Antibody** | **Information** | **Dilution** |
| --- | --- | --- |
| β-actin | Epitomics, Burlingame, CA | 1:2000 |
| TFCP2 | BD Biosciences | 1:1000 |
| HA | ABCAM | 1:400 |
| FN1 | cell signaling technology | 1:1200 |
| TJP1 | cell signaling technology | 1:1500 |
| E-cadherin | cell signaling technology | 1:1000 |
| N-cadherin | cell signaling technology | 1:1000 |
| Slug | cell signaling technology | 1:1000 |
| Snail | cell signaling technology | 1:1000 |

**Table S5. Integrate TFCP2-ChIP targets with the TFCP2-regulated transcriptome and validate the overlapped genes by real time PCR**

| Symbol | Gene ID | PEAK SCORE in ChIP in SK | SiTFCP2 vs SiNC in HepG2 | Real time PCR  (fold change relative to NC) | | | |
| --- | --- | --- | --- | --- | --- | --- | --- |
|  |  |  |  | Si HepG2 | Si BEL-7402 | TFCP2 SK-HEP1 | TFCP2 Hep3B |
| KATNAL2 | NM_031303 | 1.39 | +2.75 | +2.1 | +1.5 | -1.2 | +1.1 |
| FN1 | AB031045 | 1.29 | -2.11 | -1.8 | -2.0 | +2.7 | +1.8 |
| REEP1 | NM_080385 | 1.22 | -2.63 | -2.2 | -1.1 | +1.4 | +2.5 |
| VIPR2 | NM_002185 | 0.95 | -3.03 | -1.8 | -1.6 | - | - |
| HRK | NM_144659 | 0.81 | -2.22 | -3.2 | -2.5 | -2.7 | +1.3 |
| EID3 | XM_497365 | 0.79 | +2.20 | -2.4 | -2.8 | +1.3 | +1.4 |
| ALDH1L2 | BC001765 | 0.78 | -2.09 | -1.9 | -2.3 | -2.0 | +2.2 |
| TCTA | NM_003647 | 0.76 | +2.01 | +1.4 | -2.0 | -2.6 | -1.8 |
| ZFP36 | NM_020207 | 0.70 | -2.02 | +1.2 | +1.4 | -3.7 | -2.3 |
| FLJ40504 | BC036652 | 0.64 | -2.07 | -1.3 | -1.6 | -2.2 | -1.1 |
| CYP1A1 | NM_000499 | 0.62 | +2.83 | +1.4 | -2.2 | +2.3 | -1.7 |
| FAM81A | NM_002375 | 0.59 | -2.17 | -2.0 | -2.1 | +1.7 | +1.8 |
| MAL2 | NM_017666 | 0.58 | -2.03 | -3.1 | -2.7 | +1.5 | +1.6 |
| HSPA6 | NM_032868 | 0.56 | -2.31 | -1.2 | -3.3 | -1.6 | -2.3 |
| FRMD3 | NM_024594 | 0.55 | -2.30 | -1.8 | -2.4 | -1.9 | +2.2 |
| SETD3 | XM_926913 | 0.54 | +2.06 | -2.3 | -2.2 | -2.4 | -1.9 |
| VAV3 | NM_006113 | 0.54 | +2.73 | +2.2 | +2.7 | -1.8 | -1.7 |
| PER3 | BC008309 | 0.54 | -2.18 | -2.2 | -3.5 | +1.7 | +1.9 |
| SLC2A14 | NM_006309 | 0.54 | -2.55 | -1.3 | +3.6 | -2.6 | +1.6 |
| FAH | NM_012405 | 0.53 | -2.03 | -1.8 | -3.1 | -1.7 | +1.8 |
| SERPINB7 | AK022567 | 0.52 | -2.25 | -2.0 | -1.6 | +1.9 | +2.1 |
| RIMS4 | NM_182970 | 0.52 | +2.83 | +2.9 | +2.4 | -1.1 | -1.8 |
| RBKS | NM_001859 | 0.52 | +2.31 | +1.9 | +2.2 | -2.4 | -1.7 |
| TGFB2 | AK123361 | 0.50 | +2.03 | +2.8 | +2.3 | -1.6 | +2.2 |
| TJP1 | NM_003257 | 0.50 | +3.00 | +2.3 | +2.1 | -2.4 | -2.2 |

-, not detected, due to the too lower transcript level in the cells.
