# Supplementary material for: Characterization of genome-wide TFCP2 targets in hepatocellular carcinoma: implication of targets FN1 and TJP1 in metastasis
Source: J Exp Clin Cancer Res. 2015 Jan 22;34(1):6. doi: 10.1186/s13046-015-0121-1 (PMC4311423; doi:10.1186/s13046-015-0121-1)
Supplement: Additional file 2: Figure S1. — The effect of TFCP2 on cell migration and invasion. The TFCP2 knockdown in BEL-7402 significantly inhibited the migration and invasion of HCC cells compared with controls, whereas TFCP2 overexpression in Hep3B promoted migration and invasion. The migration and invasion assays were assessed at 24 and 48 h, respectively. The data are presented as mean ± SD of three different experiments (*P < 0.01). Figure S2. Confirmation of microarray results by qRT-PCR. Changes of selected mRNAs between the alteration of TFCP2 versus NC in HepG2 after siTFCP2. Figure S3. Biological function analysis of the TFCP2-ChIP targets in SK-HEP-1. Figure S4. Network analysis of molecule interactions from microarray data in HepG2 cells. Knowledge-based IPA network using genes in HepG2 siTFCP2-signature (genes are shown in Additional file 3: Table S3). Green/red: genes down/up-regulated in HepG2 after knockdown TFCP2. Solid lines: direct interactions, and dashed lines: indirect interactions. [file 13046_2015_121_MOESM2_ESM.doc]

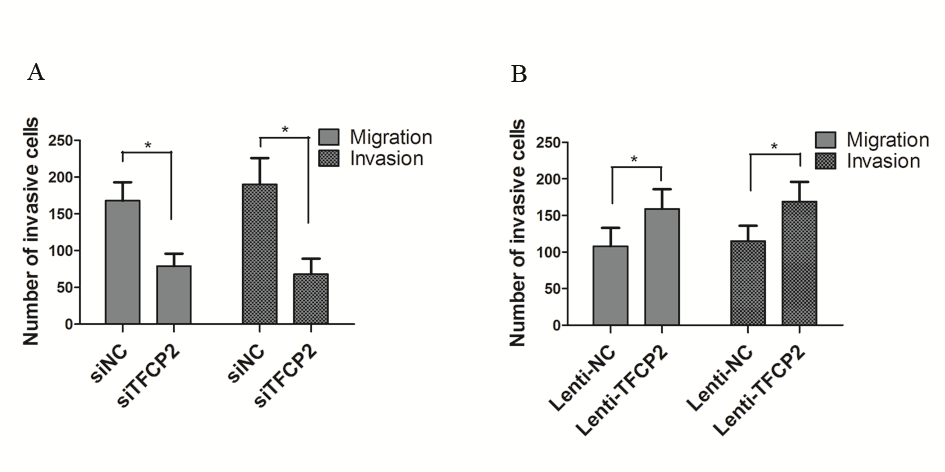


Figure S1 The effect of TFCP2 on cell migration and invasion. The TFCP2 knockdown in BEL-7402 significantly inhibited the migration and invasion of HCC cells compared with controls, whereas TFCP2 overexpression in Hep3B promoted migration and invasion. The migration and invasion assays were assessed at 24 and 48 h, respectively. The data are presented as mean±SD of three different experiments (* *P* < 0.01).


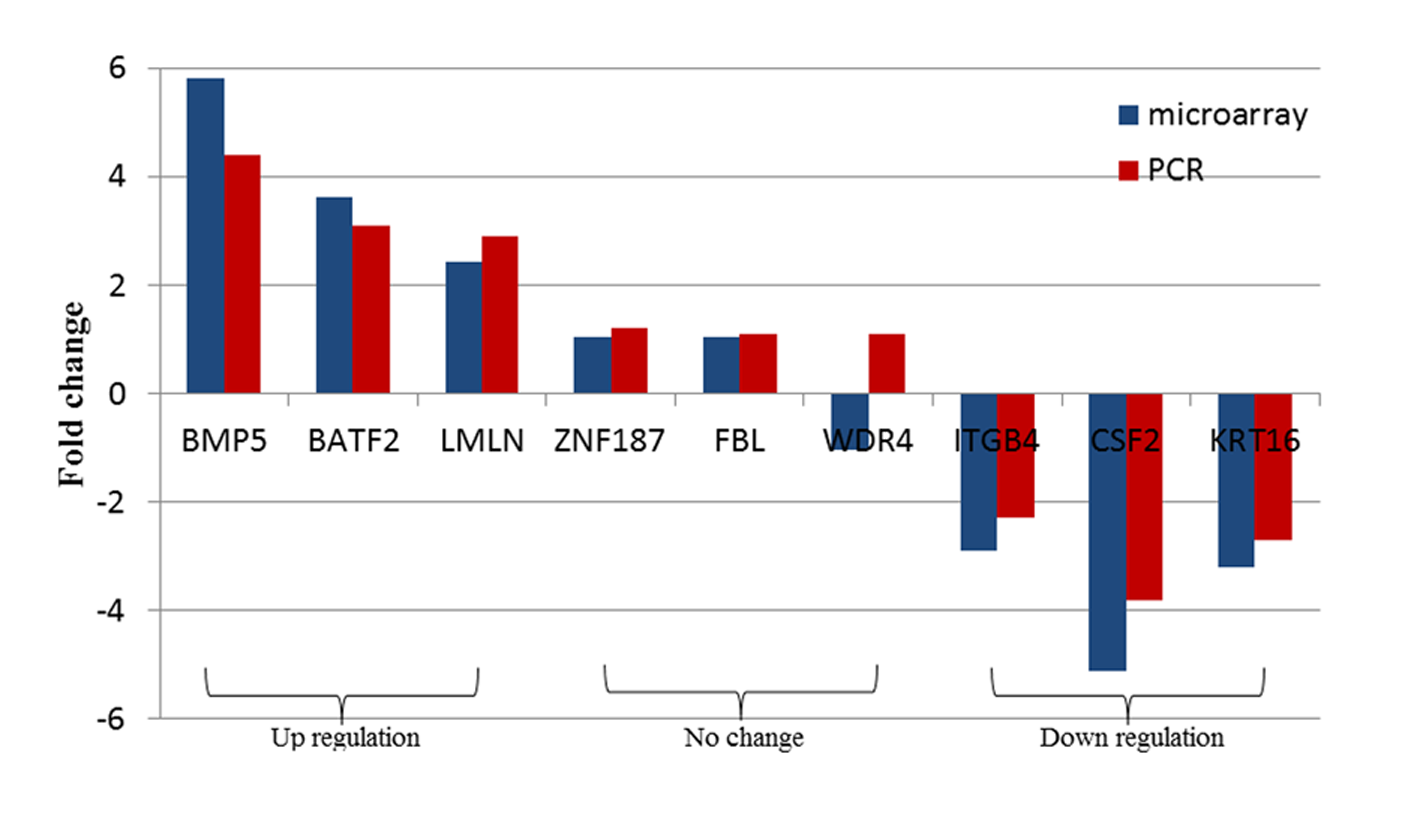


Figure S2 Conﬁrmation of microarray results by qRT-PCR. Changes of selected mRNAs between the alteration of TFCP2 versus NC in HepG2 after siTFCP2.


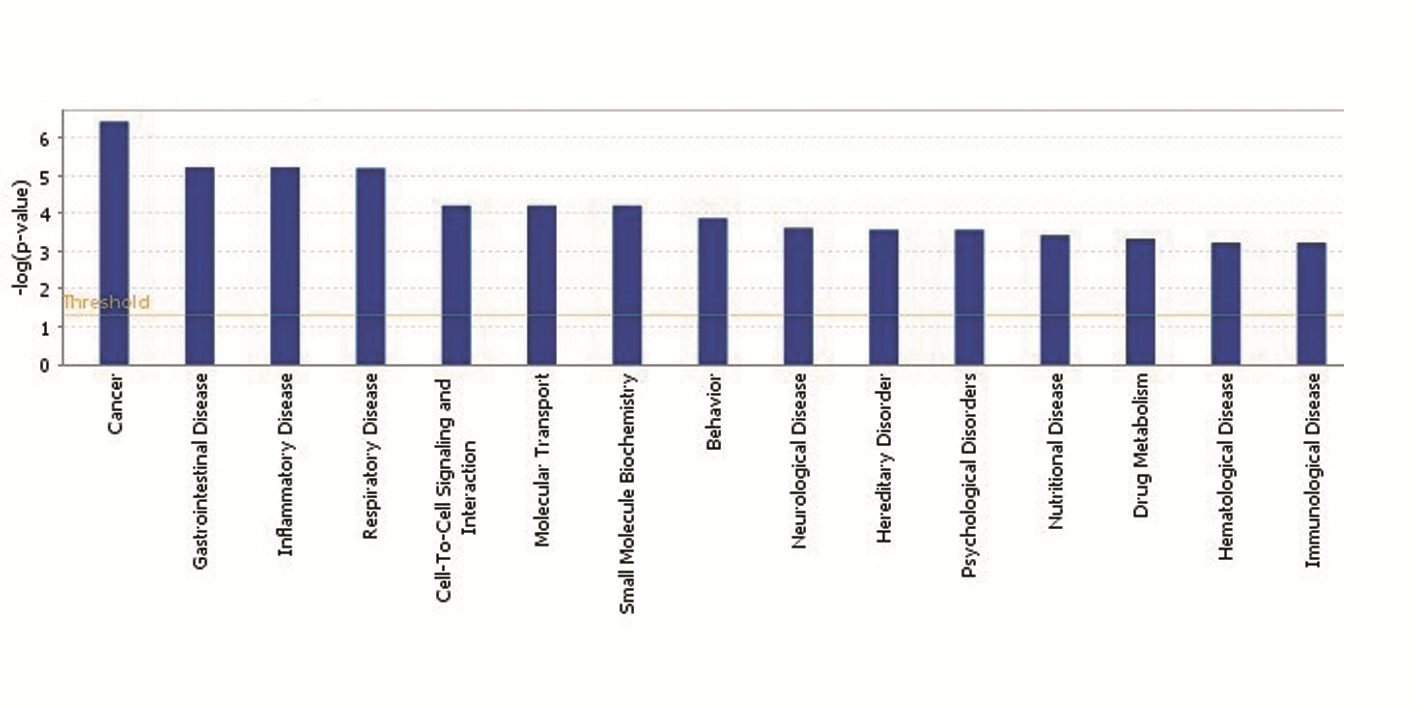


Figure S3. Biological function analysis of the TFCP2-ChIP targets in SK-HEP-1.


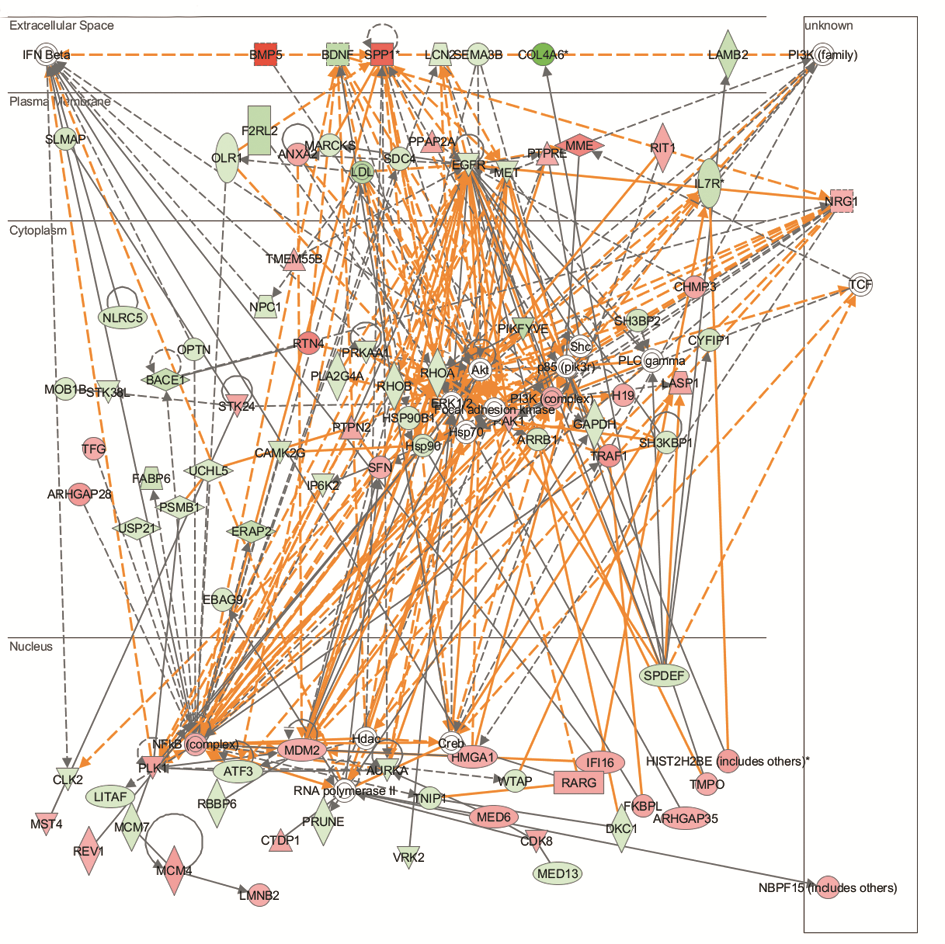


Figure S4. Network analysis of molecule interactions from microarray data in HepG2 cells. Knowledge-based IPA network using genes in HepG2 siTFCP2-signature (genes are shown in Table S3). Green/red: genes down/up-regulated in HepG2 after knockdown TFCP2. Solid lines: direct interactions, and dashed lines: indirect interactions.
